# Family Based Treatment for anorexia nervosa and reconstituted families

**DOI:** 10.1186/2050-2974-2-S1-O19

**Published:** 2014-11-24

**Authors:** Richard Litster, Tania Withington, Salvatore Catania

**Affiliations:** 1 Children's Health Queensland, Brisbane, Australia

## 

Children's Health Queensland, Child and Youth Mental Health Service (CHQ CYMHS) Family Based Eating Disorder Clinic is staffed by a multidisciplinary team trained in Family Based Treatment for Anorexia Nervosa (FBT-AN). A diverse range of families present to the clinic for treatment, including families where parents have separated and re-partnered to form reconstituted family groups with shared parenting arrangements. This is an increasingly common presentation to the Clinic regardless of child age, and presents unique challenges in the context of the Family Based Treatment Model. Whilst the Family Based Treatment Model does not focus on family etiological factors, the family unit is viewed as a crucial tool in facilitating recovery from Anorexia Nervosa and central to treatment strategies.

This presentation will discuss how Family Based Treatment is undertaken in the Clinic with reconstituted families committed to shared parenting. Case examples will be used to demonstrate the innovative strategies utilised throughout the treatment process. The task of adhering to the principals of evidence based treatment whilst responding to specific challenges for families will be examined. Critical junctures in treatment which require careful attention in order to engage both family systems whilst creating a cohesive team will be explored.

This abstract was presented in the **Service Initiatives: Child and Adolescent Refeeding and FBT** stream of the 2014 ANZAED Conference.

